# Nationwide implementation of a multifaceted tailored strategy to improve uptake of standardized structured reporting in pathology: an effect and process evaluation

**DOI:** 10.1186/s13012-022-01224-5

**Published:** 2022-07-30

**Authors:** Julie E. M. Swillens, Quirinus J. M. Voorham, Reinier P. Akkermans, Iris D. Nagtegaal, Rosella P. M. G. Hermens

**Affiliations:** 1 Scientific Center for Quality of Healthcare (IQ Healthcare), Radboud Institute for Health Sciences (RIHS), Radboud University Medical Centre, Postbus 9101, 6500 HB Nijmegen, Kapittelweg 54 (route 160), Nijmegen, The Netherlands; 2 PALGA Foundation, Houten, The Netherlands; 3 Department of Pathology, Radboud Institute for Molecular Life Sciences (RIMLS), Radboud University Medical Centre, Nijmegen, The Netherlands

**Keywords:** Clinical practice guidelines, Effect evaluation, Guideline adherence, Healthcare quality improvement, Information technology, Implementation, Oncology, Process evaluation, Standardized structured reporting

## Abstract

**Background:**

Implementation strategies are aimed at improving guideline adherence. Both effect and process evaluations are conducted to provide insights into the success or failure of these strategies. In our study, we evaluate the nationwide implementation of standardized structured reporting (SSR) in pathology.

**Methods:**

An interrupted time series analysis was conducted to evaluate the effect of a previously developed implementation strategy, which consisted of various digitally available elements, on SSR in pathology laboratories. A segmented regression analysis was performed to analyze the change in mean SSR percentages directly after the strategy introduction for pathology reporting and specific subcategories. In addition, we analyzed the change in trend in the weekly percentages after strategy introduction, also for subgroups of tumor groups, retrieval methods, and type of laboratory. The change in SSR use after the strategy introduction was determined for all pathology laboratories. We further conducted a process evaluation in which the exposure to the strategy elements was determined. Experiences of the users with all strategy elements and the remaining barriers and potential strategy elements were evaluated through an eSurvey. We also tested whether exposure to a specific element and a combination of elements resulted in a higher uptake of SSR after strategy introduction.

**Results:**

There was a significant increase in an average use of SSR after the strategy introduction for reporting of gastrointestinal (*p*=.018) and urological (*p*=.003) oncological diagnoses. A significant increase was present for all oncological resections as a group (*p*=.007). Thirty-three out of 42 pathology laboratories increased SSR use after the strategy introduction. The “Feedback button”, an option within the templates for SSR to provide feedback to the provider and one of the elements of the implementation strategy, was most frequently used by the SSR users, and effectiveness results showed that it increased average SSR use after the strategy introduction. Barriers were still present for SSR implementation.

**Conclusions:**

Nationwide SSR implementation improved for specific tumor groups and retrieval methods. The next step will be to further improve the use of SSR and, simultaneously, to further develop potential benefits of high SSR use, focusing on re-using discrete pathology data. In this way, we can facilitate proper treatment decisions in oncology.

**Supplementary Information:**

The online version contains supplementary material available at 10.1186/s13012-022-01224-5.

Contributions to the literature

- To the best of our knowledge, this is the first nationwide study evaluating the effect and feasibility of a fully digital multifaceted implementation strategy aimed at health care professionals in oncology.
- Our study showed that the various elements of the multifaceted implementation strategy, also tailored to important barriers and facilitators of the evidence-based guideline recommendation implemented, resulted in improvement of the implementation of the innovation in some but not all subgroups and feasibility results added explanations to these results on effect.
- Our study could serve as example to others who aim to improve the quality and safety by implementing and evaluating evidence-based guidelines using a fully digital multifaceted implementation strategy.

## Introduction

To continuously ensure improvement in the quality of oncological care, implementation of innovations is essential. The quality of oncological care is outlined within oncological clinical practice guidelines [1, 2]; however, on population level, there is variation in adherence to these guidelines [3–5], in particular considering the adoption of digital innovations. This situation could be improved by developing and evaluating implementation strategies [6]. Recent studies highlighted the indispensability of properly conducted evaluation studies of multi-faceted and tailored strategies, to determine how to enhance the impact of implementation strategies [7, 8]. These evaluation studies should properly examine the effect of the implemented strategy as well as the process of the implementation, to identify essential contributing factors to the success or failure of the strategy [6].

Previous research confirmed the additional value of standardized structured reporting (SSR) use compared to other types of reporting, even improving patient outcomes for certain diagnoses [9–11]. Therefore, national and international oncology guidelines advocate the use of SSR in diagnostic disciplines, such as pathology [2, 12]. Over the past years, the SSR use has increased and the number of countries adopting the International Collaboration of Cancer Reporting templates is increasing as well [13, 14]. However, differences in SSR usage are still present between countries and within countries between the reporting of tumor types, retrieval techniques, and (types of) laboratories, resulting in variation in treatment choices and therefore, patient outcomes [13, 15–18]. From our previously conducted context analyses, we retrieved barriers and facilitators for SSR implementation [19, 20].

A digitally free-offered implementation strategy, tailored to the previously found influencing factors, will have great promises for usability and practicality relative to in-person activities and even more so since the start of the COVID-19 crisis [21]. However, evidence is lacking on effectiveness and feasibility of a complete digital implementation strategy, especially for an innovation such as SSR. Therefore, we first conducted a pilot, exploring our tailored strategy on increased use of SSR among pathology laboratories for the reporting of three common groups of tumors: gastrointestinal, gynecological, and urological cancers [22]. To verify these pilot results, a large-scale national level assessment was necessary. The objective of the current study is therefore to determine effectiveness, feasibility, and combined effects of a promising multifaceted tailored implementation strategy, aimed at improving daily clinical practice at a national level. The specific objectives were as follows:

1. To determine the effect of the implementation strategy on the change in level of proportion of SSR usage and the change in linear trend in SSR usage for the following:
  a) All gastrointestinal, gynecological, and urological oncology pathology reporting
  b) Pathology reporting per tumor group (gastrointestinal, gynecological, and urological)
  c) Pathology reporting per retrieval method (biopsies and resections)
  d) Pathology reporting per type of laboratory (non-academic and academic)
2. To determine the change in the SSR use per pathology laboratory, also analyzed for the previously mentioned subgroups (b–d).
3. To determine the feasibility of the implementation strategy by the following:
  a) Determining the exposure to the different strategy elements
  b) Determining the experiences of the users of the implementation strategies
  c) Determining perceived barriers and improvements of SSR implementation
4. To determine an effect and combined effect between use of implementation strategy elements by pathology laboratories and the change in level of proportion of SSR usage and the change in linear trend in SSR usage of these pathology laboratories after strategy introduction.

## Methods

**Fig. 1:**
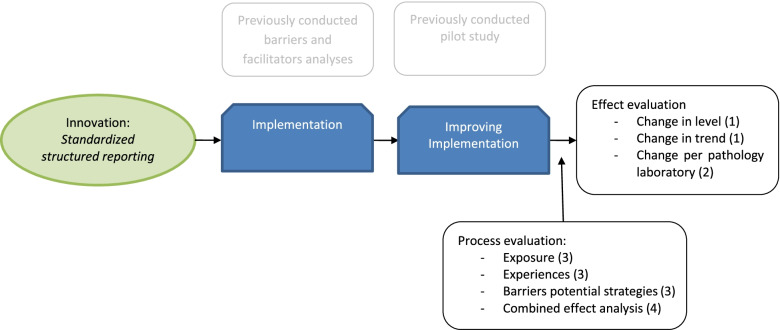
Study design

We evaluated the effectiveness of a multifaceted tailored implementation strategy and its different elements, aiming to improve the implementation of SSR, by conducting an interrupted time-series-analysis and determining the change in SSR use for all Dutch pathology laboratories. Simultaneously, we conducted a nationwide process evaluation to determine the exposure of pathology laboratories to the active strategy elements, the experiences of pathologists, pathology residents, and PALGA liaisons with the different strategy elements and perceived barriers and facilitators of SSR implementation. We also calculated an effect and combined-effect between the use of active elements and implementation effect. The study design is shown in Fig. 1. The Standards for Reporting Implementation Studies (StaRI) were used to report this implementation study [23].

### Innovation to be implemented

Pathologists could use standardized structured formats to report diagnostic findings. The use of these so-called SSR templates by pathologists is recommended in multiple Dutch [24, 25] and international guidelines [26, 27]. In the Netherlands, these templates are developed and maintained by the PALGA foundation, the Dutch nationwide pathology databank. For nineteen tumor types, a template is available [28]. The content of these templates is based on the datasets of the International Collaboration of Cancer Reporting [12] and oncology guidelines. After development, the templates are approved by the Quality and Professional Practice Committee of the Dutch Association of Pathologists [29].

### Multifaceted implementation strategy elements

The elements [22], based on previously conducted analysis of barriers and facilitators of SSR implementation, were combined into a toolbox (Additional file 1). Most barriers are related to content and readability of SSR [19, 20]. Individual elements could be used digitally on an as-needed basis. Information on the alterations made to the published small-scale tested toolbox are included in Additional file 2. The implementation strategy consisted the following:

1. Specifically designed website
2. eLearning including instruction videos on SSR usage
3. Information sheet included in SSR templates
4. Improved feedback process, including “Feedback button”
5. Audit and feedback reports showing local SSR usages
6. Communication manual

Additional information on the strategy elements, reported according to the TIDieR checklist, can be found in Additional file 3 [30]. In the communication manual, communication between laboratories, the professional organization for developing SSR templates (PALGA), and oncology-treating clinicians, by the PALGA liaison (intermediary between PALGA and the pathology laboratories), and the SSR pathologist (local key opinion leader) is described [22].

These strategy elements can be subdivided into passive elements (website, information sheet on SSR updates, and communication manual) and more active elements (eLearning, “Feedback button” and audit and feedback reports). Three editions of the audit and feedback reports on local SSR usage were sent to the pathology laboratories including data on (1) local usage in 2019 and 2020, (2) local usage in January and February 2021, and (3) local usage in March and April 2021. An example of the first edition including fictional data is provided in Additional file 4. For analysis of SSR usage on a laboratory level, all participating laboratories provided informed consent. The audit and feedback reports were sent in the first week of March (edition 1), the last week of March (edition 2), and the last week of May (edition 3).

### Nationwide dissemination of a tailored implementation strategy

**Fig. 2:**
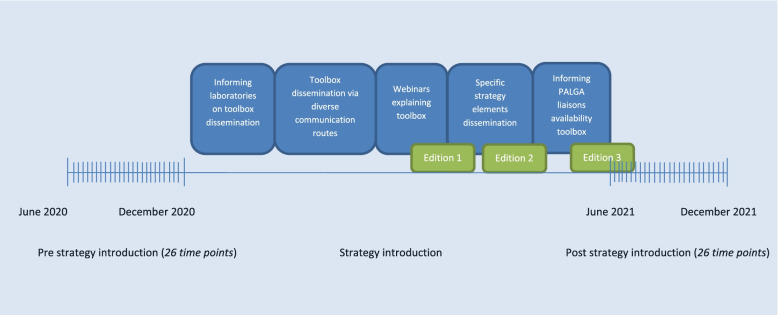
Timeline of the nationwide dissemination strategy of the tailored implementation strategy

Based on the results of our pilot [22], we determined a timeline of our national dissemination process (Fig. 2). First, all laboratory heads and PALGA liaisons were informed about the nationwide dissemination of the toolbox. Second, a description (including a hyperlink) of the toolbox was distributed via multiple national communication routes: (a) the eNewsletter of the Dutch Pathology Association, (b) the website of the Dutch Pathology Association, (c) the website of PALGA, and (d) the LinkedIn page of PALGA. The toolbox description also included a link to a 3-minute animation, showing the individual elements and instructions on how to use them properly. Third, we organized two interactive webinars including a Q&A session, to present the toolbox and to explain its different elements. Fourth, we published four different news items in the eNewsletter of the Dutch Pathology Association, each focused on a different strategy element, to emphasize the different elements, to explain to them in more detail, and to encourage the use of these elements among pathologists. Fifth, all PALGA liaisons received an e-mail including the link to the toolbox and additional information, to forward this to all pathologists in their laboratory.

### Effect evaluation

#### Study design and population

The effectiveness study consisted of monitoring the use of SSR templates (for gastrointestinal, gynecologic, and urologic oncology) in all Dutch pathology laboratories by conducting an interrupted time-series-analysis. As this study was conducted on a national level, no specific inclusion criteria were applied. The dissemination process is shown on the timeline (Fig. 2). The pre-introduction period included data from June 2020 to November 2020, the strategy introduction period ranged from December 2020 to May 2021, and the post-introduction period included data from June 2021 to November 2021, using data on SSR template use from the pathology database [31]. The unit of analysis for the effect analysis is the number of pathology reports. The selection criteria are shown in Additional file 5. In short, we included all reports, both biopsies and resections, for all suspicious malignancies (irrespective of final diagnosis) that can be reported within the available SSR templates for gastrointestinal, gynecological, and urological tumors. The colon biopsy template was excluded, since this is mandatory for the national bowel screening program and thus unaffected by the implementation strategy.

#### Data collection

The aggregated data based on continuous input from the pathology departments were evaluated for SSR usage on a weekly basis, 26 time points before and 26 time points after the dissemination period of the toolbox, as shown in Fig. 2. The primary outcome was the proportion of SSR pathology reports compared to all reporting for the specific tumor groups.

#### Data analysis

We used IBM SPSS Statistics V25.0 (IBM Corp, Armonk, NY, USA) to analyze the primary outcome among all Dutch pathology laboratories, using descriptive statistics. To analyze the effect of the nationwide implementation strategy, we conducted an interrupted time series analysis [32]. To do that, we performed a segmented regression analysis to identify if the level of proportion of SSR usage and the linear trend in SSR usage were significantly different before and after the strategy introduction. We created a data sheet including nationwide aggregated weekly SSR use among all Dutch pathology laboratories. The outcome was measured as the proportion of cases for which SSR was used in pathology reporting. The autoregressive integrated moving average (ARIMA) model including the following covariates was performed:

- Phase: binary indicator for which 0 = pre-introduction period and 1 = post-introduction period (for estimation of the change in mean percentages (level) change immediately after the strategy introduction)
- Time (in weeks): ordinal indicator expressing weeks since the start of the study (for estimation of the change in percentage with each week before the strategy introduction)
- Time since strategy introduction (in weeks): ordinal indicator expressing weeks since the introduction of the strategy (for estimation of the change in trend of the weekly percentage after strategy introduction)

All data points of the strategy introduction period had been excluded from analysis, as introduction of the strategy was occupied [33]. The impact of the strategy was also analyzed separately for subgroups (1) the reporting of gastrointestinal, gynecological, or urological oncology; (2) biopsies and resections; and (3) non-academic and academic pathology laboratories, since SSR implementation varies between tumor groups, retrieval method, and type of laboratory. We knew from our previously conducted barrier and facilitator analysis among these three tumor groups that specific influencing factors related to these subgroups of pathology reporting exist [19, 20]. Next, we added the variable “group” to test differences between the subgroups. For these tests, the gastrointestinal group, biopsy group, and non-academic laboratories group were used as reference groups. We combined the “group” variable with the other variables (strategy phase, time, and time since strategy introduction) to generate interaction variables. The autoregressive integrated moving average (ARIMA) model including all these covariances was performed. A *p* value of <0.05 was considered to be statistically significant, based on two sided tests.

We used descriptive statistics to determine the average proportion of SSR usage out of all reporting for the three tumor groups per pathology laboratory for all laboratories in the period before and after the strategy introduction. For this analysis, we also conducted a subgroup analysis per tumor group (gastrointestinal, gynecological, or urological), retrieval method (biopsies and resection), and type of laboratory (non-academic and academic).

### Process evaluation

#### Study design and population

Data on exposure rates were determined during the strategy introduction period in combination with a nationwide process evaluation, using questionnaires, to gather evidence on the feasibility of our multifaceted strategy [34, 35]. We included all pathologists and pathology residents operating in the Netherlands, being the primary users of SSR templates, and PALGA liaisons, who exchange information on SSR between pathologists of their laboratory and PALGA, and are responsible for local technical settings of SSR templates.

#### Data collection

To determine exposure to our implementation strategy elements, we combined multiple data sources, such as Google Analytics for website usage, Cuble’s statistics for eLearning usage [36], and PALGA statistics for SSR template related strategy elements. For the active strategy elements, the eLearning, the “Feedback button”, and audit and feedback reports only, we were able to collect data on a laboratory level, since due to the General Data Protection Regulation, we were not allowed to collect data such as the Internet Protocol address from website visitors. Furthermore, we performed a national survey to evaluate the process of SSR implementation, with questions about the actual use of the strategy element, user experiences regarding accessibility, content and usability, and self-reported effectiveness of mechanisms of action, more SSR usage, and better SSR usage. In addition, questions were asked on barriers of SSR implementation and their improvements. The link to the eSurvey was distributed using various channels including direct mails to PALGA liaisons and interested pathologists from existing mailing lists. In addition, calls were posted on the PALGA website, on the PALGA LinkedIn page and in eNewsletters from the Dutch Pathology Association (*n*=6) and the Dutch Pathology Residents Society (*n*=2). Information on the research question, target groups, and the anonymous data analysis was included in the introduction of the survey. Before starting the eSurvey, respondents provided informed consent for anonymous data analysis. Completing the questionnaire took 10 to 15 min for all target groups. Unanswered questions were not accepted. Recommendations of Fan & Yan [37] were used in eSurvey development and dissemination to improve eSurvey response.

#### Data analysis

Data on strategy element exposure were analyzed using descriptive statistics. For the calculation of the total exposure of the active strategy elements, the pathology laboratory not using SSR (*n*=2) were excluded, since the “Feedback button” is only accessible via the SSR templates. For the eSurvey, respondents were included when >50% of eSurvey questions were answered. We used IBM SPSS Statistics V25.0 to analyze the eSurvey results. Missing data was coded as such and excluded from data analysis. We performed descriptive statistics to determine the proportions of agreement of SSR users with the feasibility and effectiveness of the different strategy elements.

For the active elements, the eLearning, the “Feedback button”, and audit and feedback reports, we tested the differences in effect between pathology laboratories who did use, in case of the eLearning and “Feedback button”, or did receive, in case of the audit and feedback, the strategy element during strategy introduction versus laboratories who did not use or receive it. We calculated the proportion of SSR usage out of all reporting per week among the groups of pathology laboratories who did and who did not use (eLearning, “Feedback button”) or receive (audit & feedback) the specific active strategy element. The pathology laboratory not using SSR (*n*=2) were excluded from the analysis for the “Feedback button”, since this element is only accessible via the SSR templates. Besides analysis per active element, we also grouped the users of 2 or 3 active elements, 1 active element, or 0 active elements (=reference group) in three groups to test the effect of multiple active elements use on SSR usage, also excluding the two laboratories not using SSR. The autoregressive integrated moving average (ARIMA) model including all covariates (Strategy phase, Time, Time since strategy introduction, group, Strategy phase*group, Time*group, Time since strategy introduction*group) was performed. A *p* value of <0.05 was considered to be statistically significant, based on two sided tests. A comprehensive overview of the effect and process evaluation, including all outcomes, is provided in Additional file 6.

## Results

### Effect evaluation

The total included cases pre- and post-introduction were 102,587 and 110,628, respectively. We included 15,615, 57,619, and 29,623 cases pre-introduction and 16,683, 62,762 and 31,183 cases post-introduction, respectively, for gastrointestinal, gynecological, and urological oncology. The majority were resections (58.4% and 58.4%, respectively). The amount of cases (>100) per time point is included in Additional file 7. Characteristics of all Dutch laboratories (*n*=42) are shown in Table 1.

**Table 1:** Characteristics of Dutch pathology laboratories

Characteristics	*N*=42
Laboratory type	
Academic	8
Non-academic	34
Laboratory use of SSR in pre-measurement period	
Yes	40
No	2

*SSR* standardized structured reporting

#### Effect on level and trend of SSR use

**Fig. 3:**
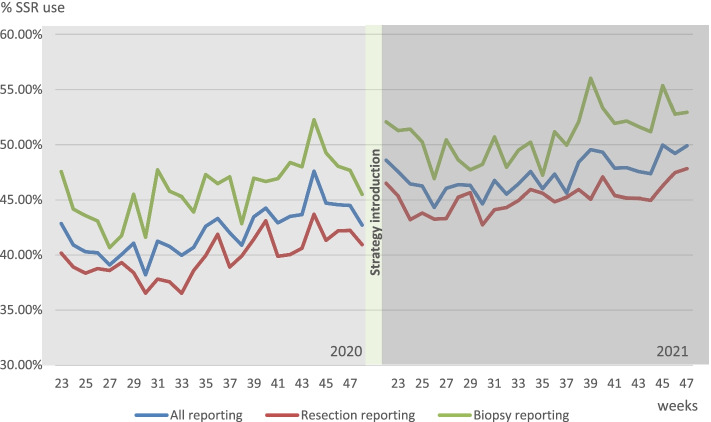
Trend in SSR use June 2020–November 2020 and June 2021–November 2021 for all pathology reporting and separately for resection reporting and biopsy reporting

Figure 3 shows the trend in SSR use for the SSR templates included. The nationwide implementation of the tailored implementation strategy caused a non-significant effect in level of SSR usage (+1.4%, *p*=.135). The change in trend of SSR use after strategy introduction compared to before was also non-significant (−0.1% per week, *p*=.277). The results of the subgroup analysis can be found in Table 2. Only the significant results of subgroups and between subgroups are reported on.

**Table 2:** Outcomes of effect evaluation and subgroups

Group pathology reporting analyzed	***n***	Subgroup	Change in level between group (%)	***p*** value	Change in level within group (%)	***p*** value	Change in trend between group (%)	***p*** value	Change in trend within group (%)	***p*** value
Tumor groups	32,298	Gastrointestinal	Ref		+2.9%	*p*=.018^A^	Ref		−0.4%	*p*=.000^a^
	120,381	Gynecological	−3.3	*p*=.045^A^	+0.1%	*p*=.970	−0.3%	*p*=.003^A^	−0.0%	*p*=.819
	60,806	Urological	+0.2	*p*=.928	+3.0%	*p*=.003^A^	+0.4%	*p*=.001^A^	0.0%	*p*=.903
Retrieval method	91,705^b^	Biopsies	Ref		+0.6%	*p*=.661	Ref		−0.0%	*p*=.589
	124,691	Resections	+1.4	*p*=.355	+2.5%	*p*=.007^A^	−.010%	*p*=.924	−0.1%	*p*=.393
Type of laboratory	188,665	Non-academic	Ref		+2.2%	*p*=.053	ref		−0.1%	*p*=.428
	24,820	Academic	+4.2	*p*=.024^A^	−2.4%	*p*=.142	+0.1%	*p*=.273	−0.2%	*p*=.064

^a^This effect was significant, based on *α*<.05

^b^As a result of a requested re-analysis, we included 2911 additional cases for this subgroup analysis only

**Fig. 4:**
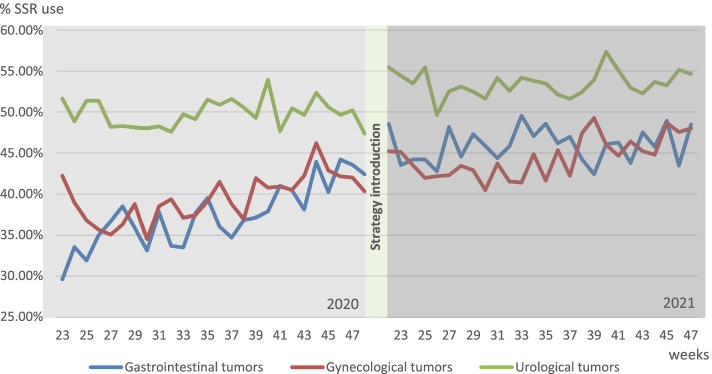
Trend in SSR use June 2020–November 2020 and June 2021–November for gastrointestinal, gynecological, and urological tumors

Trends in SSR usages per tumor group are shown in Fig. 4. The increase in level of SSR use was significant for gastrointestinal tumors: (+2.9%, *p*=.018) and urological tumors (+3.0%, *p*=.003). For gastrointestinal tumors, the slope of the trend was declining (−0.4% per week, *p*=.000). The increase in level was lower for gynecological tumors (−3.3%, *p*=.045) compared to gastrointestinal tumors. The change in trend after the strategy introduction was lower for gynecological tumors (−0.3% per week, *p*=.003) and higher for urological tumors (+0.4% per week, *p*=.001) compared to gastrointestinal tumors.

The level in SSR usage after introduction for resections only showed a significant increase (+2.5%, *p*=.007) (Fig. 3). The increase in SSR usage for non-academic laboratories is significant compared to academic laboratories (+4.2%, *p*=.024).

#### Effect on the average use of SSR in all Dutch pathology laboratories pre- and post-strategy introduction

**Fig. 5:**
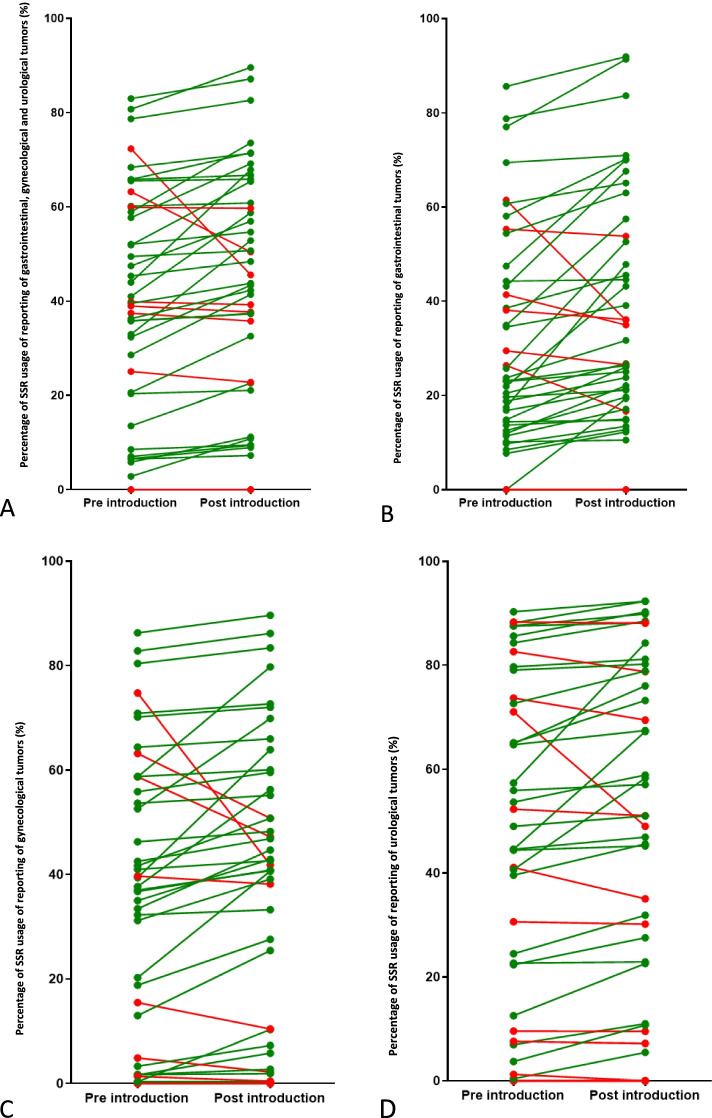
**A** National SSR usage for the pre introduction and post introduction period for all gastrointestinal, gynecological, and urological tumor reporting. Each symbol and line indicates a separate laboratory. **B** National SSR usage for the pre-introduction and post-introduction period for gastrointestinal tumor reporting. Each symbol and line indicates a separate laboratory. **C** National SSR usage for the pre-introduction and post-introduction period for gynecological tumor reporting. Each symbol and line indicates a separate laboratory. **D** National SSR usage for the pre-introduction and post-introduction period for urological tumor reporting. Each symbol and line indicates a separate laboratory

Figure 5A–D shows the average use of SSR: laboratories (33 out of 42) increased average laboratory use of SSR templates post introduction and overall, average SSR use post-introduction increased (47.3 vs. 42.3%). In the majority of laboratories, the use of SSR increased for gastrointestinal reporting (34 out of 42) for gynecological reporting (32 out of 42) and for urological reporting (28 out of 42). SSR use increased for all tumor groups: gastrointestinal reports (44.1 vs. 36.7%), gynecological reports (44.6 vs. 39.7%), and urological reports (53.5 vs. 49.9%).

**Fig. 6:**
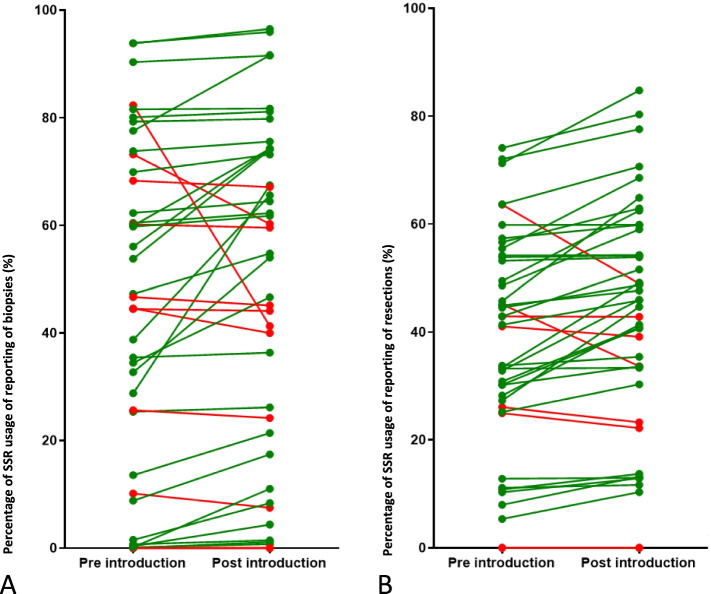
**A** National SSR usage for the pre-introduction and post-introduction period for biopsy reporting. Each symbol and line indicates a separate laboratory. **B** National SSR usage for the pre-introduction and post-introduction period for resection reporting. Each symbol and line indicates a separate laboratory

**Fig. 7:**
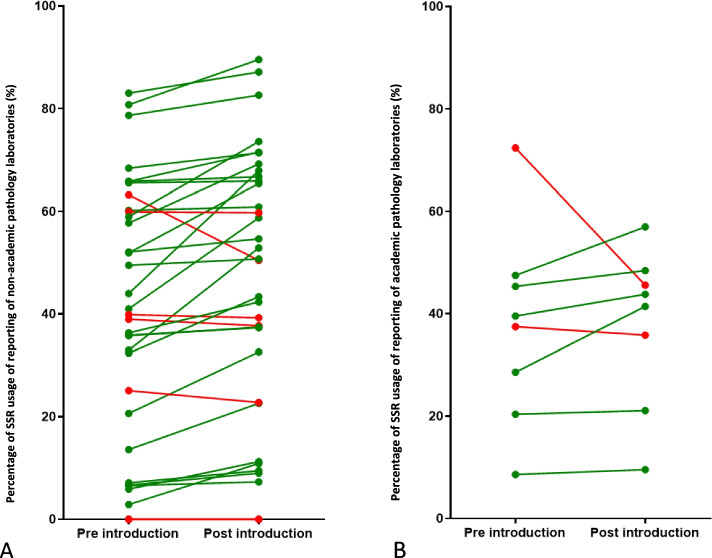
**A** National SSR usage for the pre-introduction and post-introduction period for non-academic pathology laboratories. Each symbol and line indicates a separate laboratory. **B** National SSR usage for the pre-introduction and post-introduction period for academic pathology laboratories. Each symbol and line indicates a separate laboratory

Figures 6A, B and 7A, B show the average use of SSR: there was more increase in the reporting of resection (34 out of 42 laboratories) compared to biopsies (30 laboratories). Relatively more non-academic laboratories improved (27 out of 34) compared to academic laboratories (6 out of 8).

### Process evaluation

#### Exposure

Exposure rates are shown in Table 3. We reached 33 out of 40 pathology laboratories with the active strategy elements. The “Feedback button” was the most often used active strategy element; it was used 128 times during the strategy introduction. Except for the main webpage, the webpage providing information on SSR content was the most visited webpage of the website (*n*=173). Of the 13 different eLearning courses available, the first course on the introduction of SSR templates was chosen most often (*n*=7). This was followed by the course on using fast keys within SSR templates (*n*=6), the eLearning course explaining how to properly manage the settings of SSR templates (*n*=5), and the course on how to combine two different SSR templates (*n*=5). More than half (*n*=23) of the pathology laboratories received audit and feedback reports, of which 5 pathologists of 4 different pathology laboratories replied by asking questions and reporting feedback.

**Table 3:** Overview of exposure rates per implementation strategy element during strategy introduction

Implementation strategy (element)	Active/passive	Data source	Unit of analysis	Exposure rate	
**Toolbox (*posted on webpage*)**	N/A	Google analytics	Unique visitors	108	
**Website**	Passive	Google analytics	Unique visitors	General information and benefits SSR use	194
				Overview templates and updates	173
				Development	26
				FAQ	82
**eLearning**	Active	Cuble^a^	Laboratories	9	
**Feedback button**	Active	PALGA	Laboratories	18^b^	
**Digital information sheet SSR updates**	Passive	N/A	N/A	N/A	
**Audit and feedback reports**	Active	Data agreement by e-mail	Laboratories	23	
**Communication manual**	Passive	Google analytics	Unique visitors	132	

*SSR* standardized structured reporting, *FAQ* frequently asked questions

^a^Cuble is the platform on which the eLearning was developed. This system generated weekly updates on eLearning users

^b^Feedback button used >3 times

#### Experiences

The eSurvey evaluating the process of the nationwide implementation strategy yielded 53 responses: 40 pathologists, 5 pathology residents, and 8 PALGA liaisons with an average age of 44 (range 26–66) and average years of experiences of 11 (range 1–37). Respondents were working in non-academic hospitals (59%), academic hospitals (26%), and independent laboratories (15%). The majority of the pathologists (93%) was involved in either gastrointestinal, gynecological, and/or urological oncological pathology. All were aware of SSR templates and used them in clinical practice. Of the 45 pathologists (and residents), 11 stopped using a SSR template, mostly while using the template for reporting prostate biopsies.

#### Dissemination toolbox

More than half (53%, *n*=24) of the respondents received the toolbox. Most pathologists received the toolbox via the PALGA liaison (21%, *n*=12). Three-quarter (*n*=6) of the PALGA liaison respondents received the toolbox via e-mail, and 5 of them forwarded it to pathologists within their laboratory. Half of the 30 respondents receiving the toolbox also entered the toolbox. The mean reasons for not consulting the toolbox were lack of need for assistance (23%, *n*=7) and lack of time (17%, *n* =5). Various reasons were mentioned to use the toolbox, corresponding to the widely varying rating of the toolbox with a median score of 7.0 (range 1–10).

#### Experiences with the implementation strategy elements

In Table 4, results of the eSurvey are shown. The “Feedback button” was used most frequently by the respondents (43%, *n*=19), compliant with exposure rates. All implementation strategy elements scored sufficient. The updated PALGA website was used most often (64%, *n*=7). All eLearning users (*n*=4) agreed that this could be used for resident education. Three out of 9 mentioned that the audit and feedback reports gave them new insights to improve SSR usage locally, resulting in new local agreements. The top three of the elements of respondents included all active strategy elements. Of the respondents using one or more elements, 8 out of 37 (22%) reported an effect on SSR use. Pathologists were mostly encouraged by their colleagues (9 out of 43, 21%) to start using SSR templates. Strategy elements were reviewed as very accessible (range 75–100% agreement) and usable (range 75–100% agreement). Respondents not using elements argued that they had no specific need to use it (range 48.8–61.9%) or that they were not familiar with that particular element. The latter applied less to SSR integrated elements (range 16–17.1%) compared to external elements (range 26.2–58.5%). Including the element within SSR templates was mostly chosen as an alternative method of accessibility, for both the website and communication manual (range 61.0–71.4%). Providing audit and feedback was specifically suggested as being a responsibility for the Dutch Pathology Association by 6 out of 41 respondents (15%).

**Table 4:** eSurvey results on actual use, effectiveness, and score of implementation strategy elements

Implementation strategy element		Type of element	Actual use		Effect: *More often use of SSR*		Effect: *Better use of SSR*		Recommendation score
			***n***		***n***		***n***		Median
Website SSR		Passive	11	21%	7	64%	8	73%	8 (3–10)
• FAQ			2	4%	2	100%	2	100%	8.5 (8–9)
eLearning SSR^a^		Active	4	8%	2	50%	3	75%	6.5 (4–8)
Sheet on “SSR updates”^b^		Passive	10	22%	2	20%	3	30%	7 (5–9)
Feedback button within SSR^c^		Active	19	43%	10	53%	11	58%	7 (1–10)
Audit and feedback reports local SSR use^d^		Active	9	18%	6	67%	5	56%	8 (6–10)
Key opinion leader SSR	Being	Passive	10	20%	5	50%	N/A		7 (2–10)
	Having		9	18%					
Communication manual SSR		Passive	2	4%	2	100%	0	0%	9 (8–10)
Combination of active elements		Active	23	43%	14	61%	15	65%	8 (1–10)

*SSR* standardized structured reporting, *FAQ* frequently asked questions

^a^*n*=52

^b^Could only be used by SSR template users (*n*=45)

^c^Could only be used by SSR template users (*n*=44)

^d^*n*=50

#### Barriers of SSR and potential future implementation strategy elements

Two thirds of the respondents (*n*=30) perceived barriers related to SSR use. Most perceived barriers were rigidity of SSR templates (42%, *n*=19), time needed to use SSR, and content of templates (24%, *n*=11) and SSR (20%, *n*=9), the last mostly being relevant for the reporting of ovarian cancer. Potential solutions to these barriers mentioned by pathologists and pathology residents were to improve user-friendliness, readability of SSR by using less punctuation and duplicate terminology, to integrate speech recognition and automatic transfer of clinical data.

#### Effectiveness of implementation strategy elements

There was no change in SSR usage based on the audit and feedback reports.

Pathology laboratories using the “Feedback button” ≥3 times during strategy introduction (*n*=18), significantly increased SSR use (+4.4%, *p*=.004), whereas the pathology laboratories using the “Feedback button” ≤ 3 times or not at all (*n*=24), SSR usages did not change after strategy introduction (−1.4%, *p*=.099). This was also significant different between groups (+4.2%, *p*=.016). This was a lasting effect, with no changes in trends after the introduction period (−0.1% per week, *p*=.463 vs. −0.03% per week, *p*=.610).

The use of e-learning by pathologists of specific pathology laboratories (*n*=9) did not lead to a change in SSR usage (−0.6%, *p*=.844), whereas the pathology laboratories not using e-learning (*n*=33) increased SSR usage after strategy introduction (+2.1, *p*=.019). Both groups showed no changes in trends after implementation (−0.1% per week, *p*=.827; −0.1% per week, *p*=.161). Both the change in SSR use (−3.3%, *p*=.386) as well as the change in trend in SSR use (−.064% per week, *p*=.791) were not significant between groups.

Of the pathology laboratories, 7 used 0 active strategy elements, 19 used 1 active strategy elements, and 14 used 2 or 3 active strategy elements. The use of multiple active strategy elements (2 or 3) did not lead to more a change in SSR use (+2.4%, *p*=.166) compared to using 1 or 0 active strategy elements, respectively +0.8%, *p*=.283 and +0.6%, *p*=.735), and this was not significant between groups using 1 active strategy element (+0.5%, *p*=.812) and 2 or 3 active strategy elements (+0.9%, *p*=.654) compared to the group using 0 active strategy elements. In addition, the trend in SSR per week did not change when multiple or a single active strategy element was used, respectively −0.1% per week (*p*=.513) and −0.1% per week (*p*=.306) compared to using 0 active strategy elements (−0.2% per week, *p*=.079), and this was also not significant between groups using 1 active strategy element (+0.2% per week, *p*=.250) and 2 or 3 active strategy elements (+0.1% per week, *p*=.382) compared to the group using 0 active strategy elements.

## Discussion

We aimed to improve the national implementation of SSR, by disseminating and evaluating a fully digital multifaceted tailored implementation strategy in a real-life setting. We showed that this way improvements in guideline implementation can be achieved. Effect evaluation showed a significant improvement in SSR usage for reporting for gastrointestinal and urological tumors and reporting of resections. No change was seen for the overall reporting for our three tumor groups, gynecological reporting, and biopsy reporting. A difference between academic and non-academic laboratories was present. Descriptive results showed that 33 out of 42 pathology laboratories improved their SSR use after strategy introduction. Process evaluation results for self-reported effectiveness supported this outcome, but also illustrated the additional value of other strategy elements. Our digital elements were all accessible and usable. However, integration of elements within SSR templates would increase their use. Barriers of SSR implementation, most related to rigidity, time consumption, and content of SSR template or reports, were still experienced by most pathologists and pathology residents. Effectiveness results on specific strategy elements showed that the use of the “Feedback button” was most effective in increasing SSR. Combined effect analysis showed that the use of multiple active strategy elements (2 or 3) did not lead to a change in SSR use compared to using 1 or none active strategy.

From both the effect and parallel process evaluation, we can conclude that barriers still exist, in particular for certain subgroups of reports. The presence of these persisting barriers may also explain why the use of the evaluated implementation strategy elements was still suboptimal and no combined effect was found, although dissemination of the implementation strategy was improved by incorporation of insights from our pilot study [22]. These barriers were earlier recognized [19, 20], but due to lack of time and financial resources, we could not develop specific strategies to overcome these barriers [22]. Overall, the pathology reporting for the three groups of tumors improved in the majority of the pathology laboratories, showing that small results in laboratories are possible, but the barriers for specific subgroups should be overcome to significantly improve SSR on a national level.

Our study showed the effectiveness and feasibility of a nationwide implementation of a digital innovation in pathology. This provides helpful insights to be used for other related implementation studies. Remaining barriers were rigidity of SSR templates and time needed to fill in a pathology report, already known from previous studies [19, 38, 39] and supported by the fact particularly for biopsies increase in SSR use remains relatively low. In addition, pathologists would prefer integrated implementation strategy elements. As increasingly pathology laboratories shift toward a fully digital workflow, requirements for IT development should be focused on efficient, user-friendly digital pathology workflows. For example, the use of computational pathology algorithms can potentially fill in SSR templates for biopsies, decreasing the time needed for the pathologist to fill out the SSR template. Other implementation strategy elements, such as the e-learning and audit & feedback reports, might be expanded to support the entire workflow of pathologists. Both elements could be taken up by (inter) national associations [40].

Instead of randomly choosing implementation strategy elements, we selected and developed our multifaceted implementation strategy to barriers and facilitators determined in previous studies and tested it in a small-scale setting before nationwide implementation [19, 20, 22]. Using this scientific implementation method, we were able to evaluate 6 different implementation strategy elements. However, due to organizational and technical issues, we could not test all promising strategy elements, which is one of the limitations of our study. This might explain differences in the SSR uptake between tumor groups. First, widely accepted SSR template content would benefit SSR use. By the end of the post introduction period, the first SSR template developed together with the pathology expertise group was published, setting an example for further development and improvement of other SSR templates. After establishment of a standardized governance structure on SSR development and improvement, this strategy could be evaluated, as other regions and countries implementing SSR are struggling with this as well [41]. Second, several improvements in technical aspects of the SSR template software were not developed, hampered by software design capabilities and the lack of ability to exchange discrete data with for example the hospital information system. Additionally, in case of local technical issues, having a PALGA liaison and clear communication protocols did not seem sufficient. Since pathology laboratories increasingly adopt a digital workflow, this requires dedicated staff on both a national and local level, controlling both pathology and information technology knowledge, skills, and experiences, providing pathologists with the technical assistance they need.

### Strengths and limitations

First of all, our study showed the importance of improving guideline implementation in diagnostics at a national level. Implementation decreases the variation, resulting in more standardization of diagnosis and treatment, ultimately benefiting patients. Second, in times of social distancing and also working extensively from home during the COVID-19 pandemic, our study showed that a fully digital implementation strategy facilitates continuing improvement of the implementation of a guideline recommendation. Third, international researchers and health policy makers could use our study as an example for other guideline recommendations with various adoption levels in clinical practice, for which data is already collected on a national level to be analyzed and is ready to be used to improve clinical practice. Last, by first conducting a pilot test, both the dissemination and implementation strategy were already adapted to pathologists’ needs, before scaling up to a national level.

This study also had some limitations. Relatively few research resources (both time and monetary) are available for implementation studies, resulting in a continuous circle of settling for quick and easy solutions. This does not only lower the effect of implementation studies itself, but also hampers progress in implementation science. However, we still managed to gain an effect for some subgroups of reporting by the dissemination of our implementation strategy in its current composition. Another limitation concerns the low response rate to the eSurvey, resulting in less generalizable process outcomes on experiences. However, we still gathered fruitful insights into the implementation process. A last limitation is that we could not determine the effect of all different elements on SSR usage and we could not adjust for laboratory characteristics in the combined effect analysis, since this information was unavailable. Differences between academic non-academic laboratories suggested that laboratory characteristic influence the use of strategic elements. The academic laboratories showed a decrease in SSR after induction of the implementation strategy and relatively frequent used the eLearning. This might have biased the effects of eLearning on SSR use. In addition, SSR usage was measured during the COVID-19 pandemic, in which a decline in oncology diagnoses was reported [42]. However, all measurements were conducted during this period (June 2020–November 2021) and by using a digital accessible implementations strategy, pathologists, pathology residents, and PALGA liaisons were still able to participate in this study.

## Conclusion

Nationwide SSR implementation for pathology reporting of gastrointestinal and urological cancers was improved by the implementation of a diverse pallet of digitally available implementation strategy elements, free to use for pathologists to increase their use of SSR. For other subgroups, such as the reporting of biopsies and gynecological oncology, specific barriers are known from previous analyses and these are still hampering the implementation of SSR. Since SSR, without being mandatory, is already very frequently used in pathology practice, in coming years the focus should be on how to overcome remaining barriers of SSR use in clinical practice. Moreover, now is the time for stakeholders, such as pathologists, pathology associations, pathology and cancer registries, IT, and patient associations, to closely work together to also achieve the potential benefits of SSR use by pathologists. In addition, since pathology is world leader in SSR implementation, but lags behind in other digital transitions, close collaboration with other disciplines remains on the agenda the next years, to achieve an optimal oncological diagnostic workflow. This will result in pipelines containing structured and standardized data for all diagnostic disciplines involved in oncology care, enabling improved treatment decisions.

## Supplementary Information

**Additional file 1.** Description of data: Infographic of the toolbox including all the implementation strategy elements.

**Additional file 2.** Changes to toolbox used for nationwide evaluation. Description of data: Three changes made to the previously tested toolbox.

**Additional file 3.** Overview of specifications of implementation strategy elements according to the TIDieR checklist. Detailed information on the different elements of the implementation strategy, according to the TIDieR checklist.

**Additional file 4.** Report local SSR use (audit and feedback). Report including local SSR use (fictional data included) used for the audit and feedback strategy element of the multifaceted implementation strategy.

**Additional file 5.** Selection criteria qualifying cases for effect evaluation. Applied selection criteria for the qualifying cases used in the effect evaluation.

**Additional file 6.** Overview of study outcomes. Overview of study outcomes related to the study objectives.

**Additional file 7.** Number of included pathology reports. Table including the pathology reports included in the effect evaluation, also shown for resections, and gastrointestinal, gynecological and urological tumor groups.

## Data Availability

The datasets used and/or analyzed during the current study are available from the corresponding author on reasonable request.

## Abbreviations

COVID-19: Coronavirus disease 2019
PALGA: Professional organization for developing SSR templates
ARIMA: Autoregressive integrated moving average
FAQ: Frequently asked questions
