# Supplementary figures and images for: Nationwide implementation of a multifaceted tailored strategy to improve uptake of standardized structured reporting in pathology: an effect and process evaluation
Source: Implement Sci. 2022 Jul 30;17:52. doi: 10.1186/s13012-022-01224-5 (PMC9338618; doi:10.1186/s13012-022-01224-5)

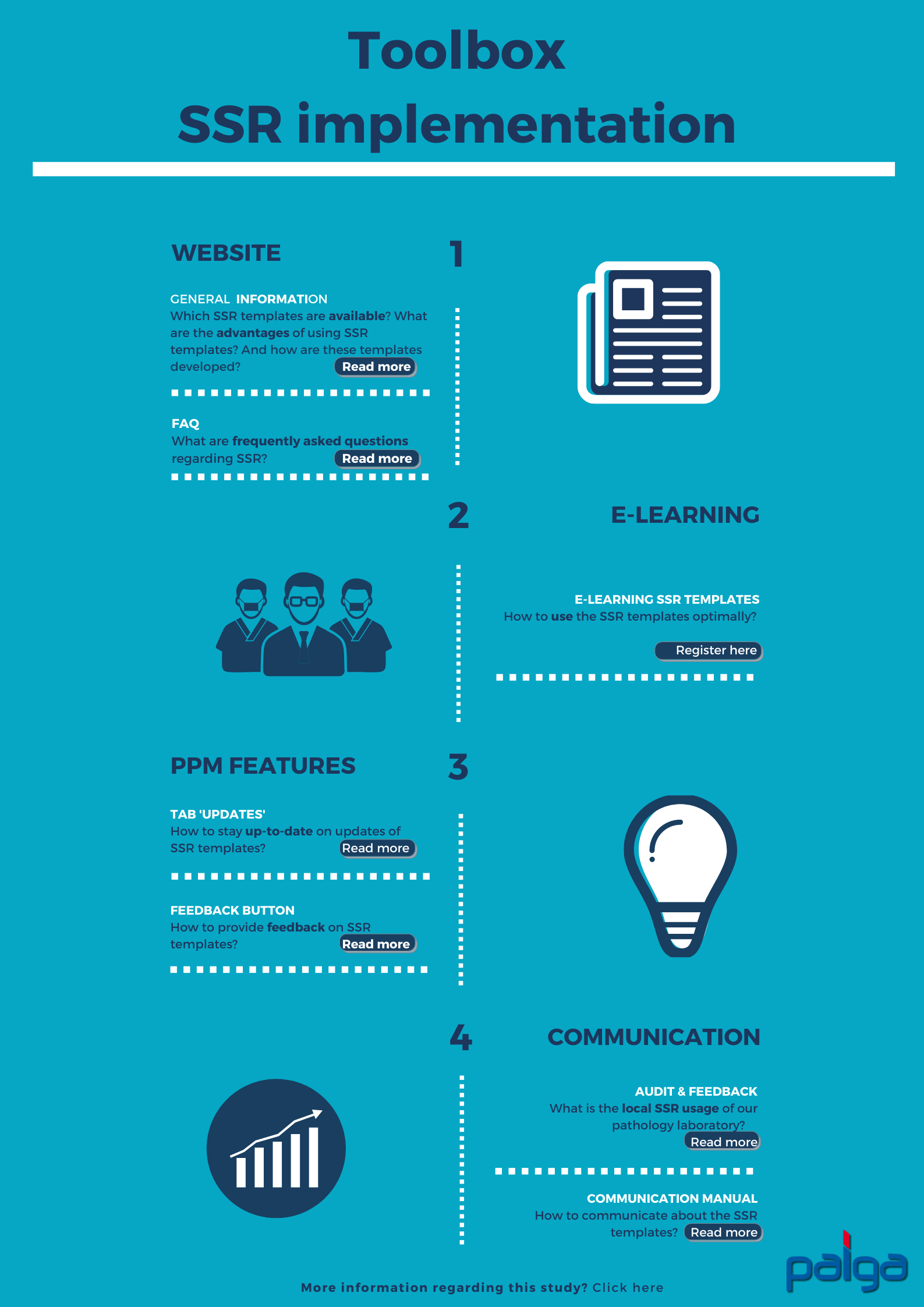

Supplement: Supplementary file 1 — Additional file 1. Description of data: Infographic of the toolbox including all the implementation strategy elements. [file 13012_2022_1224_MOESM1_ESM.png]
