# Supplementary material for: Nationwide implementation of a multifaceted tailored strategy to improve uptake of standardized structured reporting in pathology: an effect and process evaluation
Source: Implement Sci. 2022 Jul 30;17:52. doi: 10.1186/s13012-022-01224-5 (PMC9338618; doi:10.1186/s13012-022-01224-5)
Supplement: Supplementary file 2 — Additional file 2. Changes to toolbox used for nationwide evaluation. Description of data: Three changes made to the previously tested toolbox. [file 13012_2022_1224_MOESM2_ESM.pdf]

Additional file 2

Changes to toolbox used for nationwide evaluation

1. Change in method of delivery:
    a. Audit and feedback reports, 3 issues in total, were send to pathology laboratories instead of monthly telephone conversations on local SSR use
2. Regrouping of tools to clarify accessibility:
    a. The FAQ was grouped with the website, instead of the feedback procedure.
    b. The sheet on SSR updates and the Feedback Button were grouped as “PPM features”, to emphasize that these tools are integrated within SSR template software
    c. The audit and feedback reports were together with the communication manual grouped as “communication”.
3. Lay-out:
    a. The word “Step” was removed, so no consecutive order of using the tools was suggested.
    b. The “read more” buttons, including links to (information about) the tools, were highlighted in order to emphasize the ability to click on the link.
