# Supplementary material for: Nationwide implementation of a multifaceted tailored strategy to improve uptake of standardized structured reporting in pathology: an effect and process evaluation
Source: Implement Sci. 2022 Jul 30;17:52. doi: 10.1186/s13012-022-01224-5 (PMC9338618; doi:10.1186/s13012-022-01224-5)
Supplement: Supplementary file 3 — Additional file 3. Overview of specifications of implementation strategy elements according to the TIDieR checklist. Detailed information on the different elements of the implementation strategy, according to the TIDieR checklist. [file 13012_2022_1224_MOESM3_ESM.pdf]

Additional file 3. Overview of specifications of implementation strategy elements according to the TIDieR checklist (30)

	**Toolbox IMPROVING**	**1) Website**	**2)eLearning**	**3)Information sheet “SSR updates”**	**4)Feedback procedure**	**5)Audit & Feedback**	**6)Communication manual**
**WHY**							
**Justification**	Brainstorm: Previous analysis of barriers and facilitators	Brainstorm: Previous analysis of barriers and facilitators	Brainstorm: Previous analysis of barriers and facilitators	Brainstorm: Previous analysis of barriers and facilitators	Brainstorm: Previous analysis of barriers and facilitators	Brainstorm: Previous analysis of barriers and facilitators	Brainstorm: Previous analysis of barriers and facilitators
**Kok et al. (43) methods**	N.A.	Persuasive communication	Active learning	Facilitation	Participation	Feedback	Advocacy and lobbying
		Advance organizers	Modeling		Technical assistance		Modeling
			Elaboration		Advance Organizers		
**WHAT**	Dissemination	Inform	Inform and learn	Inform	Motivate to provide feedback	Provide feedback to encourage use of SSR	Communicate and support
**Materials**[A]	Infographic	Webpages	eLearning modules	Information sheet, part of all SSR templates	Feedback button, part of all SSR templates	Audit and feedback reports	Communication Manual
**Procedures**	Infographic was provided to pathologists in multiple ways	Via the toolbox (infographic) or directly via the PALGA website, pathologists had access to webpages providing information on SSR					

**WHO PROVIDED**	PALGA[B]<br>Dutch Pathology Association	PALGA	PALGA	PALGA	PALGA	Project team	PALGA
**HOW**	Online, individually	Online, individually	Online, individually	Part of SSR templates, individually	Part of SSR templates, individually	E-mail, individually or to multiple pathologists / PALGA liaison	Online, individually
**WHERE**	PALGA website<br>Dutch Pathology Association Newsletter<br>Dutch Pathology Association website	Link to website included in the IMPROVING toolbox<br>Direct access via www.palga.nl	Link to eLearning included in the IMPROVING toolbox<br>Access via www.palga.nl<br>Direct access via www.cuble.nl	Part of SSR templates	Part of SSR templates	Send via e-mail as an attachment	Link to website included in the IMPROVING toolbox<br>Direct access via www.palga.nl
**WHEN and HOW MUCH**	Freely available via internet during study	Freely available via internet during study	Freely available via internet during study	Available via SSR templates	Available via SSR templates	Three editions:<br>1) 1 March, 2021<br>2) 30 March 2021<br>3) 28 May 2021	Freely available via internet during study
**Temporality**	Use when needed	Use when needed	Use when needed	Use when needed	Use when needed	Once a month	Use when needed
**Dose**	Depends on demand of user	Depends on demand of user	Depends on demand of user	Depends on demand of user	Depends on demand of user	15 minutes	Depends on demand of user
**TAILORING**	Elements based on previously conducted barrier and facilitator analysis. Development is reported in Swillens et al. (22)						
**MODIFICATIONS**	Implementation strategy (elements) were previously tested in a pilot and modified, before being implemented and evaluated on a national level. Pilot study is reported in Swillens et al. (22)						
**HOW WELL**							

**Planned**	Individual elements could be used digitally on an as-needed basis. We evaluated the actual use of the implementation strategy (elements) on a national level. This is reported in the Method section of this article.
**Actual**	Actual use was already evaluated in our pilot study, reported in Swillens et al. (22). We also evaluated the actual use of the implementation strategy (elements) on a national level. This is reported in the Result section of this article.

[A]Materials are reported in Swillens et al. (22) and a preview of the audit and feedback report in additional file 4

[B]Professional association for developing SSR templates
