# Supplementary material for: Nationwide implementation of a multifaceted tailored strategy to improve uptake of standardized structured reporting in pathology: an effect and process evaluation
Source: Implement Sci. 2022 Jul 30;17:52. doi: 10.1186/s13012-022-01224-5 (PMC9338618; doi:10.1186/s13012-022-01224-5)
Supplement: Supplementary file 4 — Additional file 4. Report local SSR use (audit and feedback). Report including local SSR use (fictional data included) used for the audit and feedback strategy element of the multifaceted implementation strategy. [file 13012_2022_1224_MOESM4_ESM.pdf]

Additional file 4

Report

Local SSR use

[Name laboratory]

Edition 1: SSR template use 2019 – 2020

Julie Swillens, MSc
PhD student
IQ healthcare, Radboud university medical center
E: Julie.Swillens@radboudumc.nl

Q. I. Voorham, PhD
Advisor data requests
PALGA foundation
E: Rinus.Voorham@palga.nl

PALGA foundation
De Bouw 123, Houten
T: 088 0402700
E: aanvraag@palga.nl

IQ Scientific Institute for Quality of Healthcare

Radboud University Nijmegen Medical Centre

Guide

What is included in the report "Local SSR use"?

This first edition of the report "Local SSR use" shows figures of the use of the urological, gynecological and gastro intestinal oncology SSR templates over the years 2019 and 2020. This selection was made in the Dutch Cancer Society project "IMPROVING" (8281), of which this report is part of. The selection criteria can be found in Appendix 1.

Why do I receive the report "Local SSR use"?

This local report is brought to you by the Dutch Cancer Society project "IMPROVING" (8281). In this project, we have developed a set of implementation strategy elements, based on a comprehensive barriers and facilitators analysis, to improve SSR implementation. (see toolbox) In the final phase of this project, we will evaluate the implementation strategy elements at a national level. One of the tools is audit and feedback, represented by this report. Through the PALGA portal, a request was sent on 5 January 2021, to obtain permission to retrieve data regarding local SSR usage at the laboratory level.

Which information is provided within the report "Local SSR use"?

The laboratory's SSR usage is compared to the national average and, to compare usage more specifically, to the average of the type of the laboratory (non-academic/academic) to which the laboratory belongs. In this edition, you will receive information regarding SSR usage in 2019 and 2020. In the next edition, you will receive information on SSR usage in January and February 2021.

What can I use the report "Local SSR use" for?

The tables and figures included in this brief report offer pathologists in your laboratory insights into local SSR usage. This data can be shared with your colleagues to show what the current SSR usage is and where improvement opportunities exist. In this way, you can also bring the use of the SSR templates and their added value to the attention of your colleagues.

Do you have questions or remarks?

In June 2021, the implementation strategy elements, including this report, will be evaluated via a short online survey. If you have any questions, comments or suggestions for improvements at this time, we would like to hear from you. Contact details are provided on the cover page.

General information

At this page, you will find an overview of urology, gynecology and gastro enterology oncology SSR templates and the corresponding volumes on which the reporting is based. (total 2019 and 2020)

SSR template	*Total number of SSR templates of [name laboratory]*	*Total number of SSR templates including [name laboratory]*	*Total number of SSR templates of non-academic laboratories*
Appendix cancer	10[A]	642	496
Bladder/Urethra cancer	455	18.796	16.507
Cervical cancer	34	1.881	952
Colorectal cancer	360	17.210	14.933
Endometrial cancer	50	4.040	2.895
Gastric / Oesophageal cancer	22	3.158	1.526
Kidney/Pyelum/ureter cancer	4	5.029	4.075
Ovarian cancer	12	2.391	1.172
Pancreatic cancer	25	1.620	689
Prostate cancer	14	9.421	8.904
Prostate biopsy	455	38.855	34.425
Testis cancer	60	2.401	1.742

[A]*Fictional data used as illustration*

Reporting

1. **Average percentage of SSR template use [*name laboratory*] vs. average percentage of SSR template use nationally in 2019 and 2020**

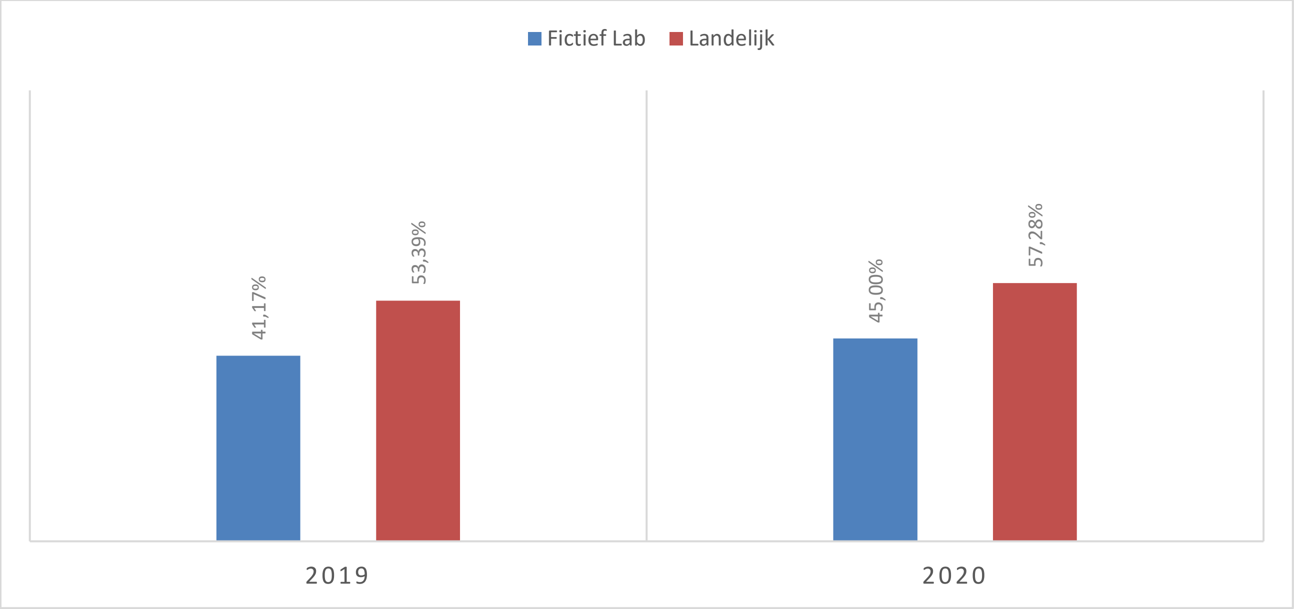

Fig. 1 *Bar graph* Average percentage SSR template use of [*name laboratory*] in 2019 and 2020 compared to the national average percentage SSR template use in 2019 and 2020 of urology, gynecology and gastro enterology oncology SSR templates

2. **Percentages of SSR template use of [*name laboratory*] vs. average percentage of SSR use nationwide in 2019 and 2020, sorted by SSR template type**

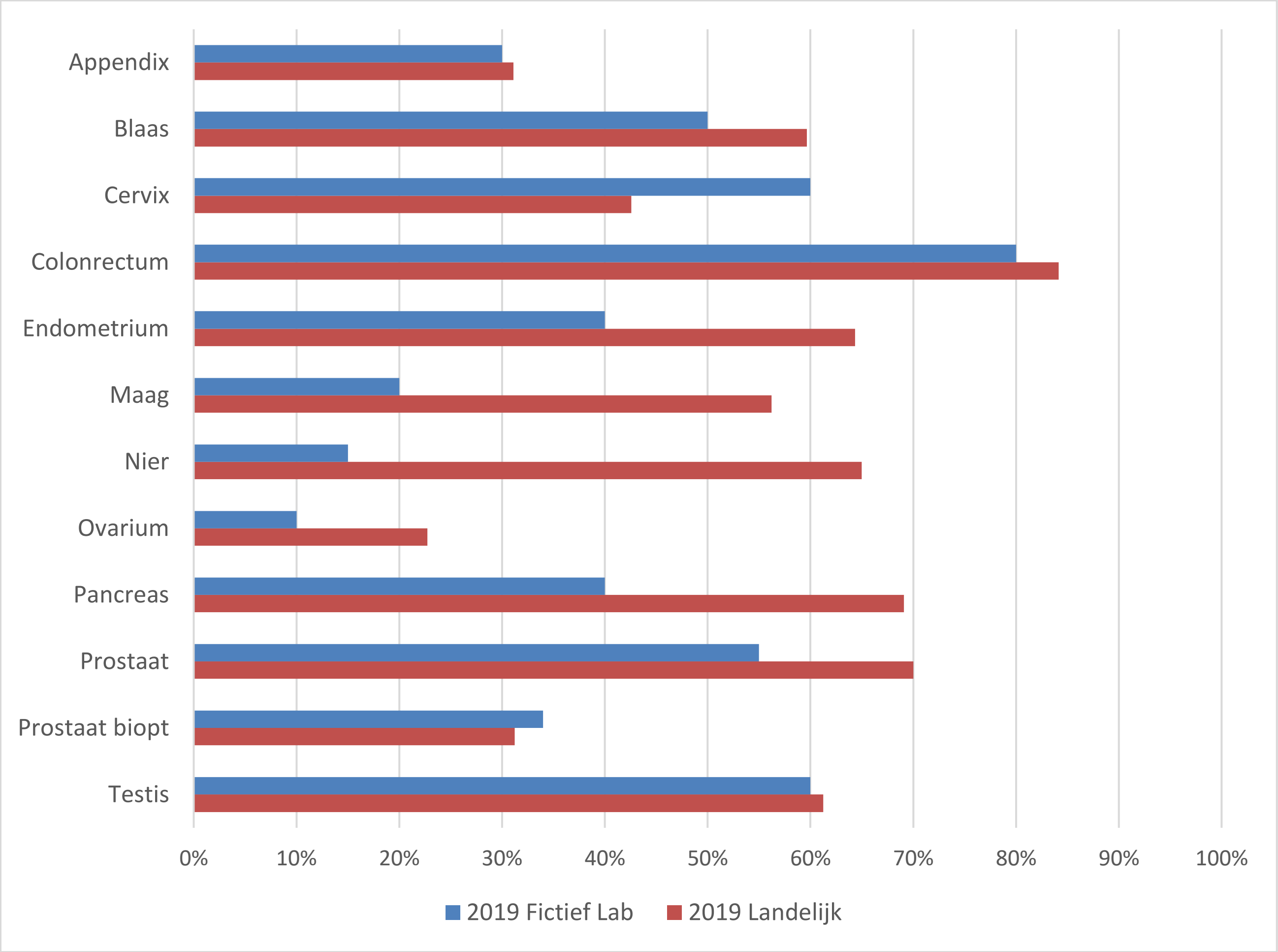

Fig. 2 *Bar graph* percentages SSR use of [*name laboratory*] in 2019 compared to average percentage SSR use nationwide, sorted by SSR template type

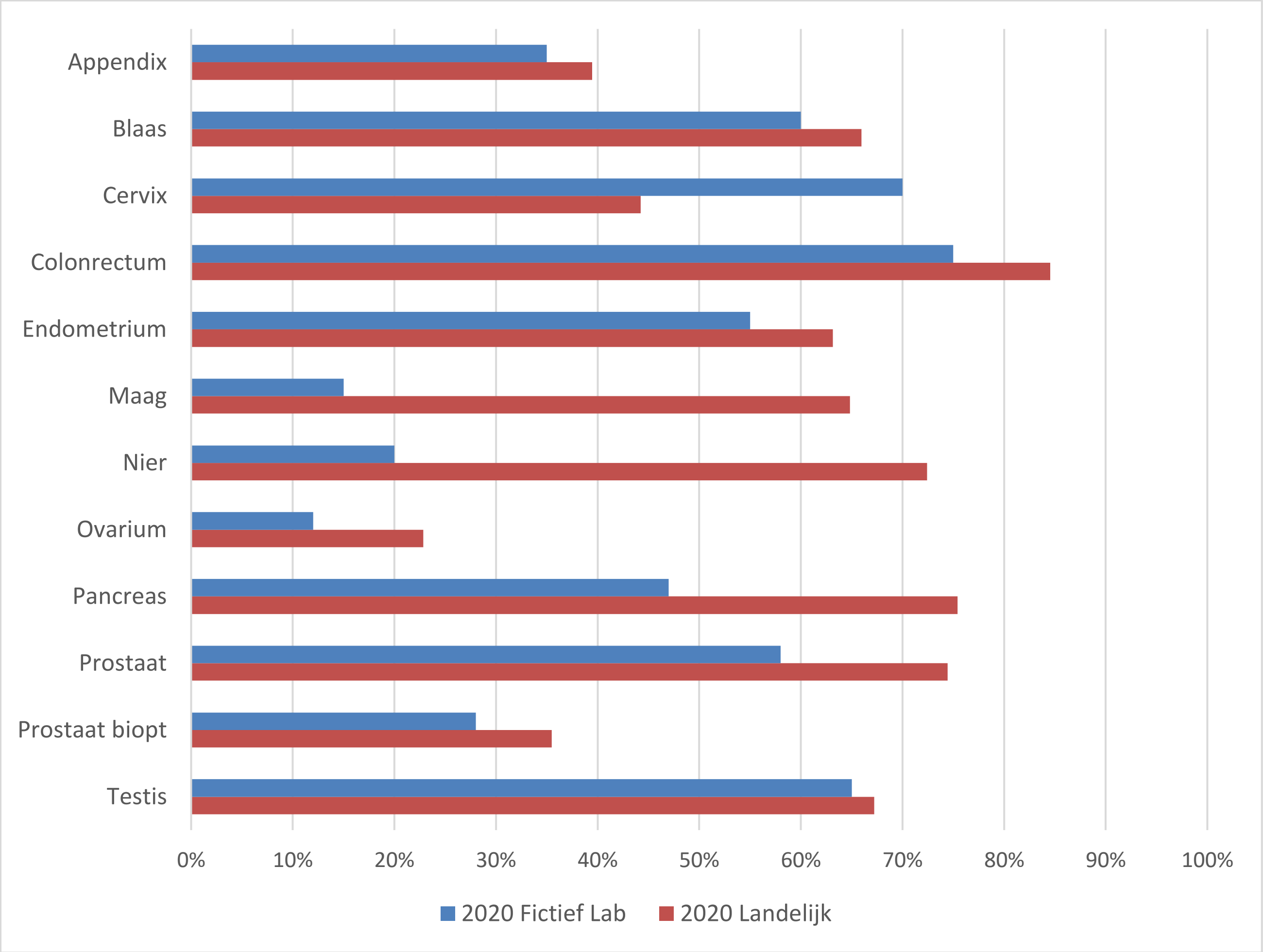

Fig. 3 *Bar graph* percentages SSR use of [*name laboratory*] in 2020 compared to average percentage SSR use nationwide, sorted by SSR template type

3. **Percentage of SSR use of [*name laboratory*] vs. the average percentage SSR use of all non-academic laboratories in 2019 and 2020, sorted by SSR template type**

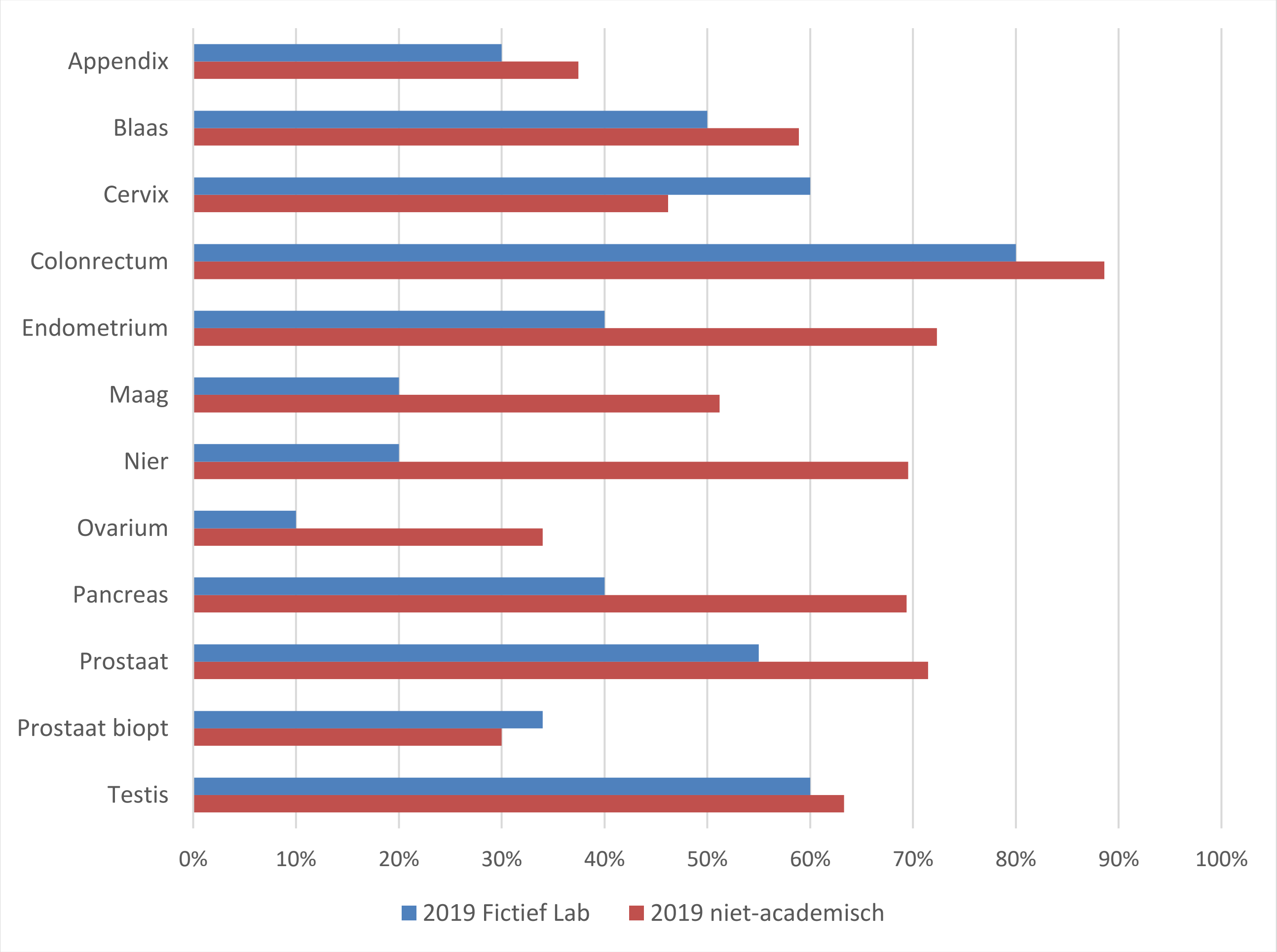

Fig. 4 *Bar graph* Percentage SSR use of [*name laboratory*] in 2019 compared to average percentage SSR use of all non-academic laboratories in 2019, sorted by SSR template type

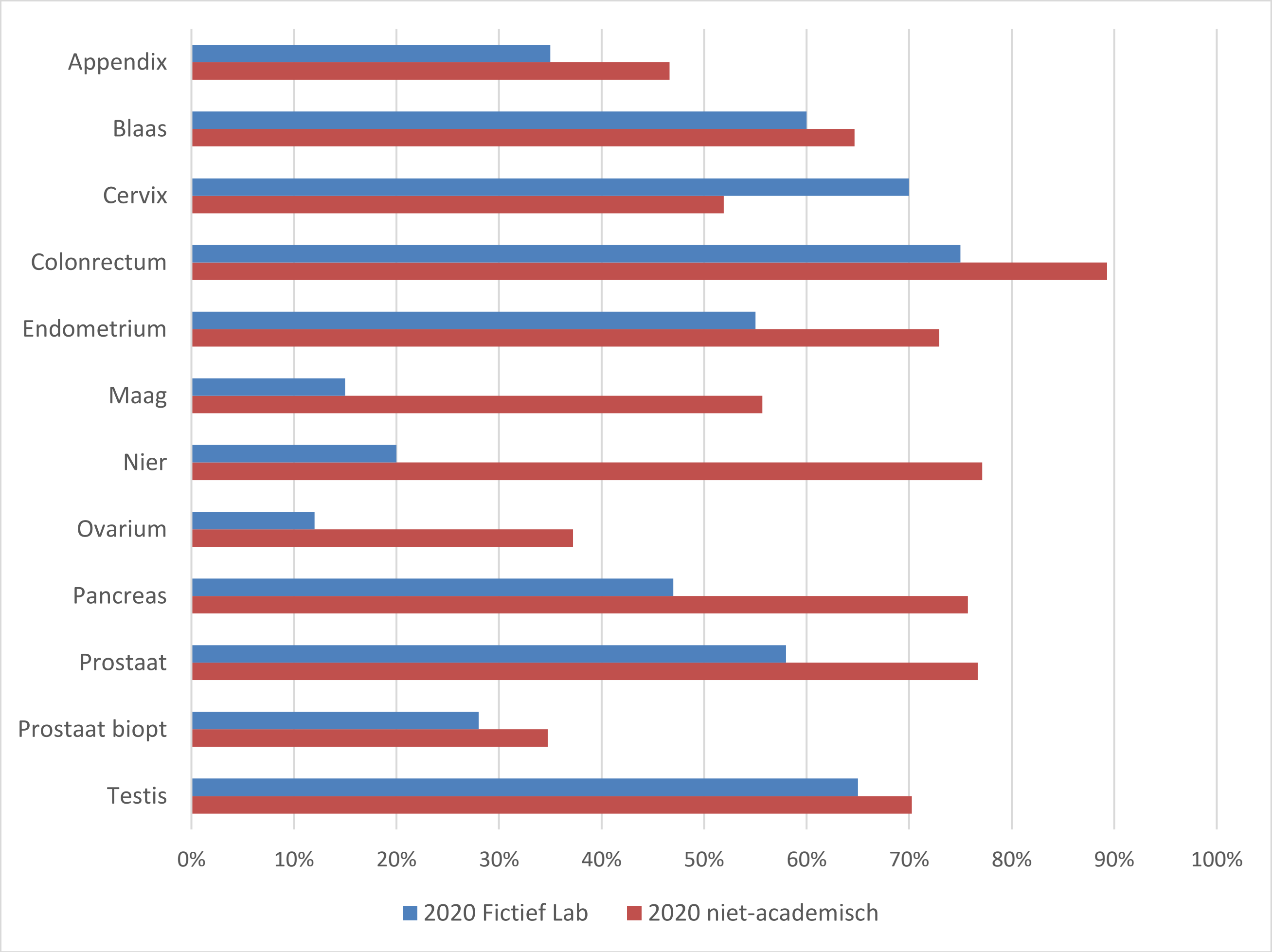

Fig. 5 *Bar graph* Percentage SSR use of [*name laboratory*] in 2020 compared to average percentage SSR use of all non-academic laboratories in 2020, sorted by SSR template type

4. Percentage of SSR use [*name laboratory*] in 2019 vs. 2020, sorted by SSR template type

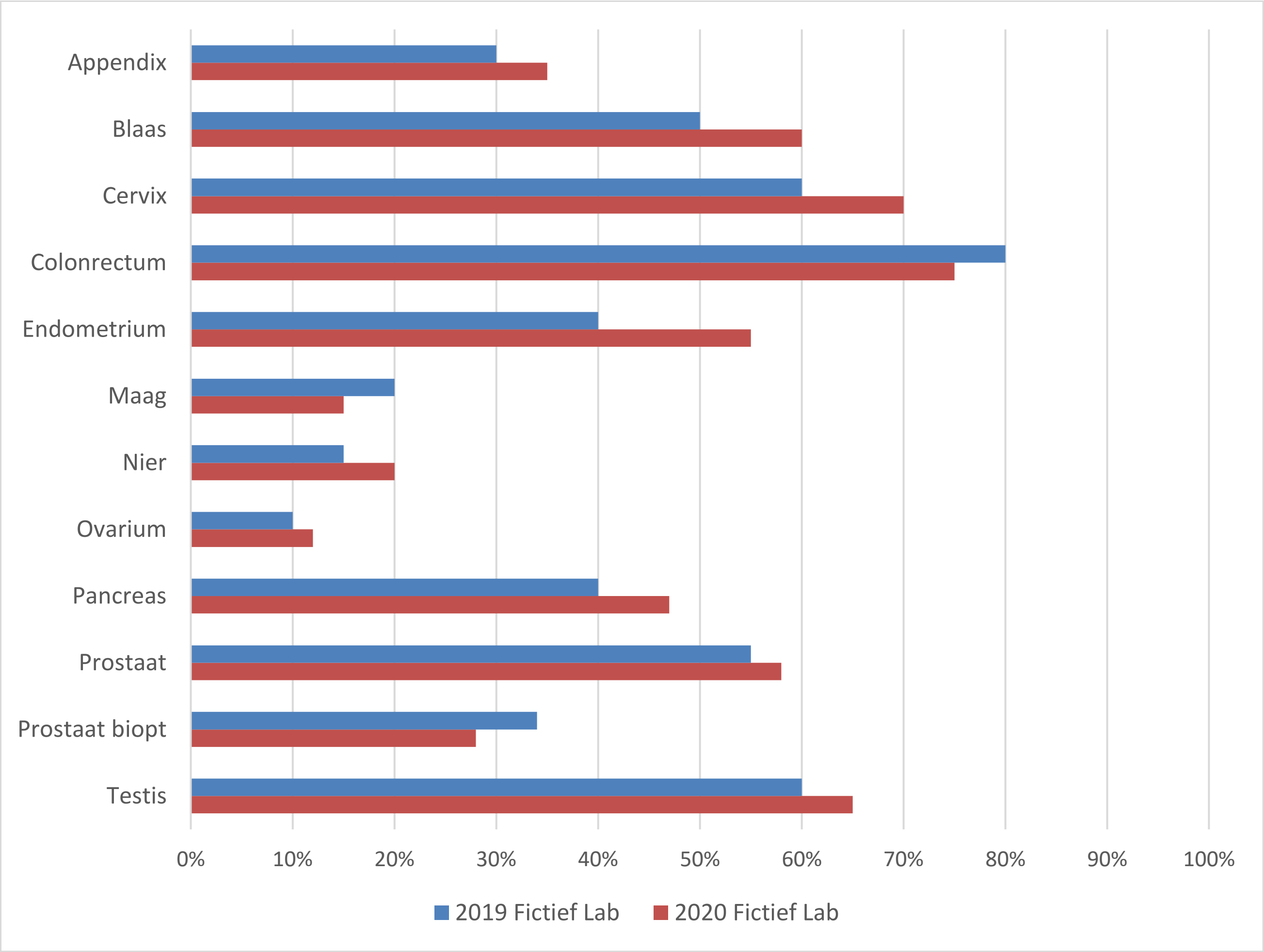

Fig. 6 *Bar chart* Percentage SSR use of [*name laboratory*] in 2019 compared to SSR use of [*name laboratory*] in 2020

Closing

We hope that this report will prompt discussion regarding SSR use within your department and within your team. In addition to this report, which provides insights into SSR use, we have developed other tools to improve SSR use:

- Website (information available SSR templates, development SSR templates and benefits of SSR use)
- E-learning (various short modules, including instructions videos, on use of SSR)
- Communication manual (divers roles and tasks of PALGA liaison and key opinion leader on SSR)
- Key components of SSR:
  - Feedback button
  - Sheet on SSR updates

All tools can be found in the toolbox. All these tools will be evaluated at the end of the project (June 2021). Your feedback is greatly appreciated!

Do you have any questions? Please contact the researcher. For questions regarding local settings, please contact your PALGA liaison.

Appendix 1 Selection of SSR use data: Retrieval terms Dutch Pathology database (PALGA)

Selection criteria qualifying cases for effect evaluation

SSR template[A]	Tissue	Retrieval technique	Diagnosis
Appendix cancer	Appendix	All resections (with curettages and small excisions)	All malignancies excl CIS
Bladder/Urethra cancer	(Bladder **OR** Urethra)	All resections (with curettages and small excisions)	All (primary) urothelial cell carcinomas
Cervical cancer	(Cervix **OR** UterusCervix)	All resections (with curettages and small excisions)	All malignancies excl CIS
Colorectal cancer	(Colon **OR** Rectum)	All resections (without curettages and small excisions)	All malignancies excl CIS
Endometrial cancer	(Endometrium **OR** UterusEndometrium)	All resections (without curettages and small excisions)	All malignancies excl CIS
Gastric / Oesophageal cancer	(Esophagus **OR** Gastric)	All resections (with curettages and small excisions)	All malignancies excl CIS
Kidney/Pyelum/ureter cancer	(Kidney **OR** Pyelum **OR** Ureter)	All resections (with curettages and small excisions)	All malignancies excl CIS
Ovarian cancer	(Ovary **OR** Tuba)	All resections (without curettages and small excisions)	All malignancies excl CIS
Pancreatic cancer	Pancreas	All resections (with curettages and small excisions)	All malignancies excl CIS
Prostate cancer	Prostate	All resections (with curettages and small excisions)	All malignancies excl CIS
Prostate biopsy	Prostate	Biopsy	All malignancies excl CIS
Testis cancer	Testis	All resections (with curettages	All malignancies excl CIS

		and small excisions)	

[A]Some tissue types have specific biopsy SSR templates used for reporting

For more information, visit: https://www.palga.nl/palga-on-line-thesaurus.html
