# Supplementary material for: Nationwide implementation of a multifaceted tailored strategy to improve uptake of standardized structured reporting in pathology: an effect and process evaluation
Source: Implement Sci. 2022 Jul 30;17:52. doi: 10.1186/s13012-022-01224-5 (PMC9338618; doi:10.1186/s13012-022-01224-5)
Supplement: Supplementary file 5 — Additional file 5. Selection criteria qualifying cases for effect evaluation. Applied selection criteria for the qualifying cases used in the effect evaluation. [file 13012_2022_1224_MOESM5_ESM.pdf]

Supplemental File 5

Selection criteria qualifying cases for effect evaluation

SSR template[A]	Tissue	Retrieval technique
Appendix cancer	Appendix	All resections (with curettages and small excisions)
Bladder/Urethra cancer	(Bladder **OR** Urethra)	All resections (with curettages and small excisions)
		Biopsy
Cervical cancer	(Cervix **OR** UterusCervix **OR** Vagina)	All resections (with curettages and small excisions)
		Biopsy
Colorectal cancer*	(Colon **OR** Rectum)	All resections (without curettages and small excisions)
Endometrial cancer	(Endometrium **OR** UterusEndometrium **OR** Adnex)	All resections (with curettages and small excisions)
		Biopsy
Gastric / Oesophageal cancer	(Esophagus **OR** Gastric)	All resections (with curettages and small excisions)
Kidney/Pyelum/ureter cancer	(Kidney **OR** Pyelum **OR** Ureter)	All resections (with curettages and small excisions)
Ovarian cancer	(Ovary **OR** Tuba)	All resections (with curettages and small excisions)
Pancreatic cancer	Pancreas	All resections (with curettages and small excisions)
		Biopsy
Prostate cancer	Prostate	All resections (with curettages and small excisions)
Prostate biopsy	Prostate	Biopsy
Testis cancer	Testis	All resections (with curettages and small excisions)

[A]Some tissue types have specific biopsy SSR templates used for reporting

In all selections molecular diagnostics and consultation/revision are excluded

* excluding (hyperplastic) polyp, adenoma, dysplasia, Inflammatory bowel disease, inflammation, infection, necroses, endometriosis, Hirschsprung, stoma, pouch, ischemia, cyst, lymphoma
