# Supplementary material for: Nationwide implementation of a multifaceted tailored strategy to improve uptake of standardized structured reporting in pathology: an effect and process evaluation
Source: Implement Sci. 2022 Jul 30;17:52. doi: 10.1186/s13012-022-01224-5 (PMC9338618; doi:10.1186/s13012-022-01224-5)
Supplement: Supplementary file 6 — Additional file 6. Overview of study outcomes. Overview of study outcomes related to the study objectives. [file 13012_2022_1224_MOESM6_ESM.pdf]

Additional file 6

**Objective**	**Analysis**	**Outcome**	**Unit of analysis**	**Time(s) of measurement**
Evaluate effect of nationwide implementation	Interrupted time series analysis	Proportion of SSR usage out of total pathology reporting per week	Pathology report	26 times before strategy introduction,26 times after strategy introduction
Subgroup analysis	*Subgroup*	*Groups*		
	Tumor types	Gastrointestinal		
		Gynecological		
		Urological		
	Retrieval method	Biopsies		
		Resections		
	Type of laboratory	Non-academic		
		Academic		
Evaluate SSR use per laboratory	Descriptive statistics	Average proportion of SSR usage out of total pathology reporting	Pathology report	2 times (Before and after strategy introduction)
Subgroup analysis	*Subgroup*	*Groups*		
	Retrieval method	Biopsies		
		Resections		
	Tumor types	Gynecological		
		Gastrointestinal		
		Urological		
Evaluate actual use of implementation strategy elements	Descriptive statistics	Binary outcome use/not use of implementation strategy element	Pathology laboratory	1 time (during strategy introduction)
User experiences	Descriptive statistics	Accessibility<br>Content<br>Usability	Pathologist, pathology resident or PALGA liaison	1 time (after strategy introduction)
Self-reported effectiveness	Descriptive statistics	More use of SSR<br>Better use of SSR	Pathologist, pathology resident or PALGA liaison	1 time (after strategy introduction)
Combined effect	Combined effect analysis	Proportion of SSR usage out of total pathology reporting per week for three groups (2-3 tools used, 1 tool used and 0 tools used)	Pathology report	26 times before strategy introduction and 26 times after strategy introduction
Subgroup analysis	*Subgroup*	*Groups*		
	Feedback button	Users		
		Non-users		
	eLearning	Users		
		Non-users		
	Audit & feedback	Users		
		Non-users		
