# Supplementary material for: Nationwide implementation of a multifaceted tailored strategy to improve uptake of standardized structured reporting in pathology: an effect and process evaluation
Source: Implement Sci. 2022 Jul 30;17:52. doi: 10.1186/s13012-022-01224-5 (PMC9338618; doi:10.1186/s13012-022-01224-5)
Supplement: Supplementary file 7 — Additional file 7. Number of included pathology reports. Table including the pathology reports included in the effect evaluation, also shown for resections, and gastrointestinal, gynecological and urological tumor groups. [file 13012_2022_1224_MOESM7_ESM.pdf]

Additional file 7

Number of included pathology reports

**Weeks**	**Total reporting**	**Resections**	**Gastroinstinal**	**Gynecological**	**Urological**
2020					
23	3311	1981	537	1835	939
24	4079	2405	605	2334	1140
25	3953	2346	639	2152	1162
26	4014	2448	617	2214	1183
27	4069	2435	643	2258	1168
28	3927	2332	613	2200	1114
29	3790	2177	582	2094	1114
30	3376	2063	613	1776	987
31	3248	1975	556	1734	958
32	3384	1967	555	1867	962
33	3264	1938	576	1781	907
34	3673	2099	571	2082	1020
35	4006	2388	620	2278	1108
36	4109	2450	646	2286	1177
37	4173	2493	654	2253	1266
38	4200	2480	657	2317	1226
39	4196	2467	579	2363	1254
40	4308	2582	626	2401	1281
41	4405	2482	600	2496	1309
42	3958	2257	539	2224	1195
43	4020	2243	559	2365	1096
44	4194	2284	578	2460	1156
45	4320	2431	604	2479	1237
46	4124	2301	597	2367	1160
47	4416	2495	629	2566	1221
48	4340	2530	620	2437	1283
Total	*102,857*	*60,049*	*15,615*	*57,619*	*29,623*
2021					
22	4602	2710	680	2633	1289
23	4667	2781	728	2603	1336
24	4599	2670	683	2613	1303
25	4469	2684	701	2470	1298
26	4534	2670	741	2544	1249
27	4572	2710	710	2600	1262
28	4417	2595	700	2467	1250
29	3874	2294	581	2184	1109
30	3637	2120	597	1969	1071
31	3487	2042	588	1930	969
32	3386	1979	554	1830	1002
33	3460	2082	565	1895	1000
34	3929	2262	633	2272	1024
35	3919	2391	556	2245	1118
36	4342	2520	686	2484	1172
37	4330	2584	683	2434	1213

Weeks	Total reporting	Resections	Gastroinstinal	Gynecological	Urological
38	4453	2530	626	2559	1268
39	4486	2630	660	2582	1244
40	4513	2609	579	2623	1311
41	4508	2619	659	2569	1280
42	3893	2263	582	2195	1116
43	4319	2512	648	2458	1213
44	4838	2831	697	2818	1323
45	4683	2613	668	2725	1290
46	4327	2524	621	2444	1262
47	4384	2417	557	2616	1211
Total	*110,628*	*64,642*	*16,683*	*62,762*	*31,183*
